# CircRNA-006258 Sponge-Adsorbs miR-574-5p to Regulate Cell Growth and Milk Synthesis via EVI5L in Goat Mammary Epithelial Cells

**DOI:** 10.3390/genes11070718

**Published:** 2020-06-28

**Authors:** Meng Zhang, Li Ma, Yuhan Liu, Yonglong He, Guang Li, Xiaopeng An, Binyun Cao

**Affiliations:** College of Animal Science and Technology, Northwest A&F University, Yangling 712100, Shanxi, China; dreamfxr@163.com (M.Z.); mali112800@nwafu.edu.cn (L.M.); lyhzzero@126.com (Y.L.); heyonglong@nwafu.edu.cn (Y.H.); liguangdky@163.com (G.L.); anxiaopengdky@163.com (X.A.)

**Keywords:** *EVI5L*, miR-574-5p, circRNA-006258, milk synthesis, dairy goats

## Abstract

The development of the udder and the milk yield are closely related to the number and vitality of mammary epithelial cells. Many previous studies have proved that non-coding RNAs (ncRNAs) are widely involved in mammary gland development and the physiological activities of lactation. Our laboratory previous sequencing data revealed that miR-574-5p was differentially expressed during the colostrum and peak lactation stages, while the molecular mechanism of the regulatory effect of miR-574-5p on goat mammary epithelial cells (GMECs) is unclear. In this study, the targeting relationship was detected between miR-574-5p or ecotropic viral integration site 5-like (*EVI5L*) and circRNA-006258. The results declared that miR-574-5p induced the down-regulation of *EVI5L* expression at both the mRNA and protein levels, while circRNA-006258 relieved the inhibitory effect through adsorbing miR-574-5p. *EVI5L* blocked the G1 phase and promoted the S phase by activating the Rab23/ITGB1/TIAM1/Rac1-TGF-β/Smad pathway in GMECs. By increasing the protein expression of Bcl2 and reducing the protein expression of Bax, *EVI5L* promoted cell growth and inhibited apoptosis. The activation of the PI3K/AKT–mTOR signaling pathway promoted the production of triacylglycerol (TAG) and β-casein in GMECs. The circRNA–006258/miR-574-5p/EVI5L axis could regulate the cell growth and milk synthesis of GMECs by sponge-adsorbed miR-574-5p. These results would provide scientific evidence for precision animal breeding in the industry of dairy goats.

## 1. Introduction

MicroRNAs (miRNAs) are a series of small non-coding RNA gene products containing 18–22 nucleotides. Recently, researchers found that miRNAs can bind to 3’ untranslated region (3’ UTR) of target messenger RNAs (mRNAs) and negatively regulate gene expression by transcriptional or translational inhibition [1,2,3]. MiR-574-5p are implicated in the pathophysiology of many tumors, including breast cancer, colorectal cancer and other oncoma [4,5,6]. In addition, miR-574-5p is a very important regulator for cell migration and proliferation [7,8,9]. Based on our laboratory sequencing data, miR-574-5p is differentially expressed in the colostrum and peak lactation periods [10]. MiR-574-5p targets MAP3K9 and reduces the secretion of β-casein and triglycerides through PI3K/AKT–mTOR pathways in goat mammary epithelial cells (GMECs) [11].

Circular RNAs (circRNAs), a kind of endogenous non-coding RNA (ncRNA) with a closed-loop structure, were discovered 40 years ago and are synthesized by a back-splicing event of protein-coding mRNAs that occurs during post-transcriptional processes [12]. At present, many experiments have proved that circRNAs are modulators in numerous malignancies, and the process of cell growth and invasion [13,14,15]. At the present stage, considerable studies have found that circRNAs act as ‘miRNA sponges’ and bind proteins to affect a variety of biological processes [16]. Nevertheless, whether circRNAs participate in the GMEC growth and progression remains to be clarified, and the underlying mechanisms of circRNAs are not expounded syllabify in GMECs.

The Ecotropic viral integration site 5-like (*EVI5L*) is a member of the EVI5 family. The EVI5 family belongs to a small subfamily of the Tre-2/Bub2/Cdc16 (TBC) domain-containing proteins [17,18]. Comparing mouse with human, the EVI5 family has two prominent domains: a Tre-2/Bub2/Cdc16 (TBC) domain which is related to the proteins that act as GTPase activating proteins (GAPs) for the Rab family GTPases at the N-terminal half, and a coiled-coil region bearing homology to the structural maintenance of chromosomes (SMC) family of ATPases at the C-terminal half [19,20]. Some reports showed that EVI5 might target Rab11 in cytokinesis [19,21], while *EVI5L* acts on Rab10 and Rab23 in cytosolic [22]. *EVI5L* takes part in primary cilium formation through binding Rab23 and the high expression of *EVI5L* significantly reduces primary cilium formation by more than half. Lim’s tests revealed that *EVI5L*, a putative Rab23 GAP, regulated the signal way-sonic hedgehog (Shh) signaling during mouse growing [23]. For mouse embryos, *EVI5L* expression was increased in Tgif1, Tgif2-null embryos and in double-null mouse embryo fibroblasts (MEFs) [20]. At present, all of the study of *EVI5L* is about the primary cilium formation, and reports about other functions of *EVI5L* are few. 

*EVI5L* was chosen for two reasons: (1) the 3’-UTR of *EVI5L* has the special nucleotide sequence which attached to the seed sequence of miR-574-5p; (2) *EVI5L* enigmatically plays roles in cell cycle progression, cytokinesis, and cellular membrane traffic [18]. In our previous work, the ncRNA library was obtained by bioinformatics prediction method. On account of the circRNA-006258 was a significant difference, and we selected it for the future studies. The regulation of circRNA-006258/miR-574-5p/*EVI5L* was detected by establishing an in vitro culture system of GMECs. Our purpose was to explore a circRNA–miRNA–mRNA network involved in the regulation of mammary epithelial cell growth and milk synthesis, and to reveal its molecular mechanism for the regulation of the lactation performance of dairy goats.

## 2. Materials and Methods

### 2.1. Mammary Gland Sample Collection

All the experimental animals in this study were raised in accordance with the announcement NO.5 of the Ministry of Agriculture, and the program of the animal experiment was approved by the animal experiment program application review committee of Northwest A&F University.

In this study, three Guanzhong dairy goats (3 year old, female) at 90 days postpartum during peak lactation were chosen from a local experiment station in Northwest A&F University of China. 

The research animals were anesthetized by the intramuscular injection of 150 mg phenobarbital sodium. After 30 min, using a scalpel, incision of 1 cm was cut in the middle of mammary gland, removing about 1 cm^3^ of mammary gland tissue, before suturing and sterilizing the wound. After 1 week, the surgical line was removed, and all the animals recovered. The mammary tissue samples were placed in phosphate buffer saline (PBS) and transferred immediately to the laboratory. There were 100 μg/mL streptomycin and 100 μg/mL penicillin in the PBS [22,24].

### 2.2. Cell Culture 

Firstly, fresh udder tissues were washed several times until the solution was pellucid and without milk. Secondly, the tissues were cut about 1 mm^3^ and were washed again with PBS. The smaller tissues were cultured in DME/F-12 medium (Gibco, Waltham, MA, USA) with 10% fetal calf serum (Gibco, Waltham, MA, USA), 5 μg/mL insulin, 100 U/mL penicillin and streptomycin (Gibco, Waltham, MA, USA), 10 ng/mL epidermal growth factor 1 (EGF-1, Gibco, Waltham, MA, USA) at 37 °C in a humidified atmosphere with 5% CO2. After about 1 week, the purified GMECs were received.

### 2.3. Vector Construction

Some genes were predicted can bind miR-574-5p by using Target Scan (http://www.targetscan.org/). The *EVI5L* CDS sequence (XM_018050954.1) of the goat were predicted by the NCBI genome website. Firstly, the 3′ UTR fragment of EVI5L was cloned and building the plasmids of EVI5L with psiCHECK2 vector was to obtain WT-EVI5L-psiCHECK2. The fragment was cloned by PCR with the total RNA that was isolated from the GMECs. The specialized pMD™19-T vector (TaKaRa, Beijing, China) was used to get a high-efficiency PCR product and obtained the complete sequence. *EVI5L* was inserted into the pcDNA3.1 vector (Thermo Fisher, Shanghai, China) so that the restriction enzyme cutting site was Kpn I and Not I. *EVI5L* (pcDNA3.1) primer information for the vector construction: Forward: CACTAGAGATGGCGAGCCCCACTCTG. Reverse: CTGGGTACCTCAGTTGTCCAGGCCCTGGCT.

### 2.4. Luciferase Assay

Before the luciferase assay, the psiCHECK-2 vectors (Addgene, Watertown, USA) of the *EVI5L* and circRNA-006258 were built. The miRNA-574-5p target sites were found from the former studies [11]. Primers of wild psiCHECK-2 vectors were designed and synthesized in the 3’ UTR of *EVI5L* and circRNA-006258 with special restriction enzyme sites: Xho I and Not I. The primers were used for psiCHECK-2 vectors in Table S1. Then, when the GMECs had a density of 50,000 cells/well in 48-well plates, 0.33 mg psiCHECK-2-*EVI5L* and psiCHECK-2-circRNA-006258 were cotransfected, respectively, with 10 pmol miRNA-574-5p mimics or inhibitors into cells. After 24 h, renilla and firefly luciferase activities were measured using thermo scientific varioskan flash (Thermo scientific, Waltham, MA, USA) by the Dual-Glo luciferase assay system (Promega, Madison, WI, USA).

### 2.5. Transfection and RNA Extraction

The cells were cultured in 6-well plates and the density of every well was 80–90%. Using Lipofectamine RNAiMAX Reagent (Invitrogen, Carlsbad, CA, USA) transfected into GMECs with NC, inhNC, miR-574-5p mimics/inhibitors, si-*EVI5L* (small interfering RNA of EVI5L) (GenePharma, Shanghai, China), si-circ-006258 (GenePharma, Shanghai, China), pcDNA3.1, pcDNA3.1-*EVI5L* plasmids, after 24 h or 48 h, the cell samples were collected for further experiment. Total RNA was isolated using Trizol reagent (Invitrogen, CA, USA) [25]. RNA concentration and purity were evaluated with Agilent 2100 Bioanalyzer (Agilent Technologies, PaloAlto, CA, USA) and the RNA was stored at −80 °C. Table S2 provides the sequences of *EVI5L*, circRNA-006258 and miR-574-5p.

### 2.6. Reverse Transcription and Real-Time PCR

Reverse transcription was conducted according to the introduction of PrimeScript RT reagent Kit with gDNA Eraser (TAKARA, Beijing, China) [26]. SYBR Premix Ex Taq II (TAKARA, Beijing, China) was used to quantify the mRNA standards of disparate genes and the statistics were analyzed with the CFX Connect Real-Time PCR Detection System (Bio-Rad, Hercules, CA, USA). The validated primers used for RT-qPCR are listed in Table 1. The RT-PCR conditions were: 95 °C for 10 min and then 40 cycles at 94 °C for 15 s, 60 °C for 30 s, followed by 72 °C for 30 s. U6 and β-actin were applied to the internal controls for *EVI5L* and miR-574-5p mRNA [27]. Each experiment was independently repeated at least three times. The relative expression level was calculated by the *N* = 2^−ΔΔCt^ method [28]. The analyses were performed in triplicate.

### 2.7. Western Blot

Cells transfected for 48 h were collected, then the cells were lysed by RIPA (Radio Immunoprecipitation Assay) lysis buffer (Bioteke, Beijing, China) and centrifuged at 4 °C for about 15 min (12,000 ×g). The total protein would go through a series of experiments: 12% SDS-polyacrylamide gel electrophoresis (SDS-PAGE) separated the different protein, and the gels were moved to PVDF (Polyvinylidene fluoride) membranes (Merck Millipore, MA, USA). The PVDF membranes were sealed with 10% skimmed milk powder for 2 h at room temperature. After the membranes were washed three times with Tris-buffered saline plus Tween 20 (TBST), and they were then incubated with the primary antibodies for 4 h, and the mouse or rabbit antibodies for 2 h. All of the main antibodies are shown in Table 2. Subsequently, all the proteins were tested by Quantity One program (Bio-Rad, CA, USA).

### 2.8. Cell Cycle, Proliferation and Apoptosis Assay

Cell viability and proliferation were tested as previously described with CCK-8 (ZETA™ life, San Francisco, CA, USA) and Edu (Ribobio, Guangzhou, China) [24,29]. The GMECs were cultivated in 96-well plates. After 24, 48, and 72 h transfection, 20 μL CCK8 solution from each well was added to 20 μL CCK8 solution. About 2 h later, using the Epoch microplate reader (Biotek, Winooski, VT, USA), the absorbance at 450 nm was measured. After 24 h transfection, the GMECs were treated with 50 Μm for about 2 h and then DAPI (4’,6-diamidino-2-phenylindole) was added to the mix solution for about 15 min at 37 °C. Then, the cells were washed in PBS three times. A fluorescence microscope was calculated for taking the cell images. The ratio of EdU positive cells (EdUstaining cells/the total of cells) was calculated and each experiment had three independent replicates.

Cell cycle staining Kit (SeaBiotech, Shanghai, China) and the flow cytometer was performed to detect the cell cycle. The cells were harvested and were washed three times with PBS. Then, 75% ethanol in PBS fixed the cell overnight at −20 °C. The apoptosis of the GMECs was tested with the flow cytometry method (FCM) and an Annexin V-FITC PI staining apoptosis assay kit (SeaBiotech, Shanghai, China). There were three replicates per condition for every group.

### 2.9. Determination of β-Casein and Triglyceride Analysis 

After 24 h transfection, the cell-free supernatants and lysates were obtained and were used to test β-casein and the triglyceride analysis. β-casein and the triglyceride analysis of GMECs were carried out with an Enzyme-linked immunosorbent assay (ELISA) kit (Tongwei, Shanghai, China) and a triglycerides quantitative assay kit (Applygen, Beijing, China), respectively [11]. 

### 2.10. Statistical Analysis

SPSS 19.0 (Beijing, China) were used to processes all the data present in the experiment. The data could not just be the same and were revealed as the means ± SE (standard error) of the three independent experiments The differences of each group were analyzed using one-way and two-way analysis of variance (ANOVA), then a Bonferroni post-hoc correction for all group comparisons was performed, considered statistically significant at * *p* < 0.05 and ** *p* < 0.01. 

## 3. Results

### 3.1. EVI5L Is One of Target Genes of miR-574-5p in GMECs

**Figure 1:**
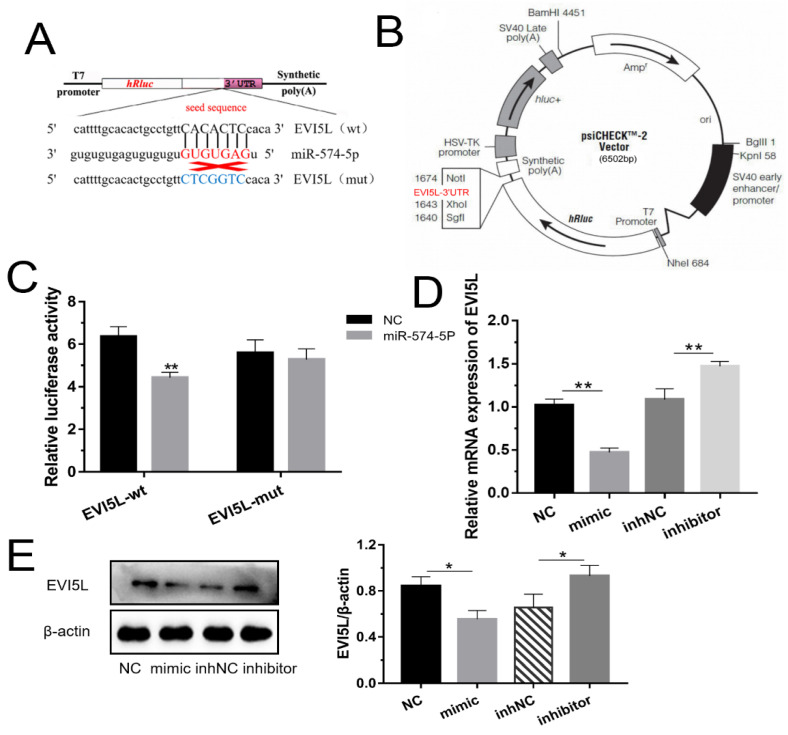
MiR-574-5p-targeted and down-regulated *EVI5L.* (**A**) *EVI5L* presents a complementary sequence (GCS), 3′ untranslated region (3′ UTR), binding the seed sequence of miR-574-5p. (**B**,**C**) Contribution vector to test the luciferase expression and the result of the luciferase assay. (**D**) The analysis result about the mRNA expression of *EVI5L* by RT-qPCR. (**E**) Western blot analysis of *EVI5L* expression after 48 h transfection with miR-574-5p mimics, inhibitors, NC and inhNC in goat mammary epithelial cells (GMECs). WT-*EVI5L*-WT, WT-*EVI5L*-psiCHECK2; *EVI5L*-mut, MUT-*EVI5L*-psiCHECK2; NC: negative control; inhNC: inhibitor negative control. ** means significant difference (*p* < 0.01), * means significant difference (*p* < 0.05).

The 3’ UTR of *EVI5L* has the special nucleotide sequence which could be specifically attached to miR-574-5p (Figure 1A,B). From Figure 1C, the groups co-transfected with WT-*EVI5L*-psiCHECK2 and miR-574-5p mimic significantly attenuated luciferase activities compared with the experiment groups that were co-transfected with WT-*EVI5L*-psiCHECK2 and negative control (NC). There was no significant change in the co-transcriptional group of miR-574-5p and MUT-*EVI5L*-psiCHECK2. The results of mRNA detection (Figure 1D) indicated that miR-574-5p mimics apparently down-regulated *EVI5L* compared to NC; inversely, the *EVI5L* expression with miR-574-5p inhibitors transparently reduced in contrast with the inhibitor negative control (inhNC). Western blot (Figure 1E) analysis also demonstrated that the miR-574-5p mimics reduced the protein of *EVI5L*, while the inhibitor-treated group had an opposite performance compared with the inhNC. To sum up, miR-574-5p played a demotivated role for the expression of *EVI5L* on mRNA and protein levels.

### 3.2. EVI5L Arrested G0/G1 Phase and Promoted S Phase in GMECs

**Figure 2:**
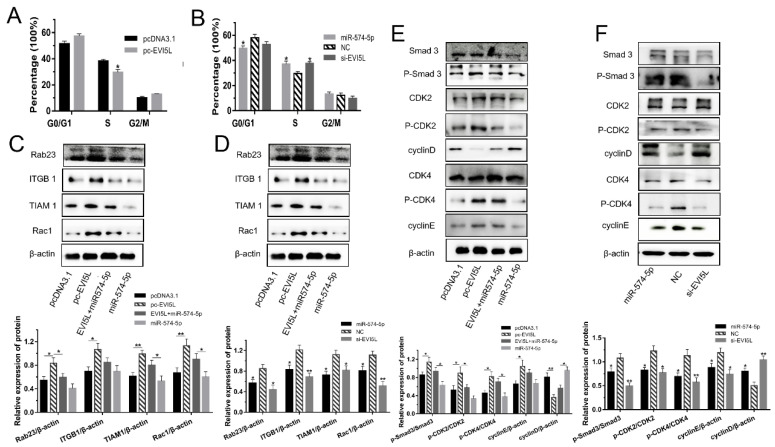
*EVI5L* caused the G0/G1 phase arrest and promoted the S phase in the GMECs. (**A**) Pc-*EVI5L* and pcDNA3.1 were transfected in GMECs and the cells were cultured for about 24 h before collection, then the cell cycle was examined by FCM. (**B**) FCM analyzed the cell cycle of the GMECs transfected with miR-574-5p, si-*EVI5L*, and NC. (**C**,**D**) *EVI5L* affected the protein expression of the Rab23/ITGB1/TIAM1/Rac1 pathway in the WB assay. (**E**,**F**) Western blot analysis used to assess the effect of *EVI5L* on the TGF-β/Smad pathway in GMECs. FCM: flow cytometry analysis; pc-*EVI5L*, overexpression *EVI5L* plasmid; pcDNA3.1: control group; NC: negative control; si-*EVI5L*: small interfering RNA of EVI5L; *EVI5L +*miR-574-5p: pcDNA3.1-*EVI5L*+miR-574-5p. ** means significant difference (*p* < 0.01), * means significant difference (*p* < 0.05).

As shown in Figure 2A,B and Figure S1A,B, for S phase, the number of GMECs in the control group (pcDNA3.1) was more than in the overexpression *EVI5L* plasmid (pc-*EVI5L*). However, the groups transfected with miR-574-5p, si-*EVI5L* and the negative control (NC) indicated that the percentage of cells in the G0/G1 phase of miR-574-5p was less than the NC; as miR-574-5p and si-*EVI5L* had more cells in S phase than the NC, observably. Therefore, it turned out that *EVI5L* caused the G0/G1 phase arrest and promoted the S phase in GMECs.

The WB experiment was performed to assess the molecular mechanism of *EVI5L* on cell cycle regulation in GMECs. Pc-*EVI5L* increased the protein expression of ITGB1, TIAM1, Rac1 comparing with pcDNA3.1, and compared miR-574-5p or si-*EVI5L* with the negative control (NC), but the WB result was opposite for ITGB1, TIAM1 and Rac1 (Figure 2C,D). *EVI5L* also influenced the pathway of TGF-β/Smad (Figure 2E,F). Compared with pcDNA3.1, pc-*EVI5L* improved the total protein and phosphorylation of Smad3, CDK2, CDK4 and promoted the cyclinE protein, but decreased the protein of cyclinD. Quite the contrary, miR-574-5p and si-*EVI5L* had different works. In short, *EVI5L* regulated the cell cycle via the Rab23/ITGB1/TIAM1/Rac1-TGF-β/Smad pathway in GMECs.

### 3.3. EVI5L Promoted the Growth of GMECs 

**Figure 3:**
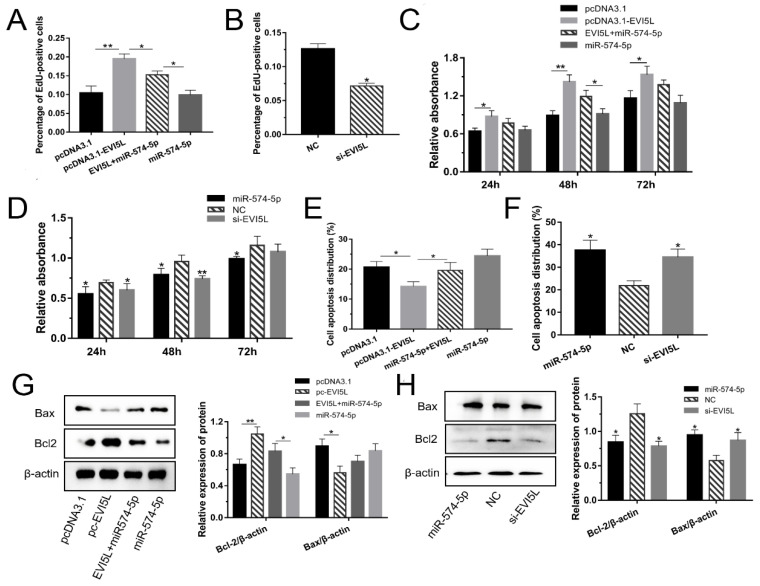
*EVI5L* promoted the growth of GMECs. (**A**,**B**) EdU was used to analyze cell proliferation when the GMECs were cultured for about 24 h after transfection.(**C**,**D**) Using CCK-8 to test the activity of GMECs which were cultured for 24, 48 and 72 h after transfection. (**E**,**F**) Flow cytometry was performed to analyze cell apoptosis after transfecting for about 24 h. (**G**,**H**) WB assay was conducted to investigate the protein expression of Bcl2 and Bax in the GMECs. Pc-*EVI5L*: overexpression *EVI5L* plasmid; pcDNA3.1: control group; NC: negative control; si-*EVI5L*: small interfering RNA; miR-574-5p*+EVI5L*: miR-574-5p+pcDNA3.1-*EVI5L*. ** means significant difference (*p* < 0.01), * means significant difference (*p* < 0.05).

As shown in Figure 3A and Figure S1C, the overexpression of *EVI5L* made the quantity of cells better than with pcDNA3.1; comparing with the miR-574-5p+pcDNA3.1-*EVI5L* group, the number of cells in the pcDNA3.1-*EVI5L*-treated group was upgraded; while the cells in the group of miR-574-5p mimics decreased. We speculated that *EVI5L* could promote GMEC proliferation. The trials of NC and si-*EVI5L* validated this speculation (Figure 3B and Figure S1D). The result of CCK-8 illustrated in Figure 3C,D indicated that *EVI5L* activated the GMECs and weakened the function of miR-574-5p in the viability of GMECs. Besides, flow cytometry was performed to analyze cell apoptosis (Figure 3E,F and Figure S1E,F). *EVI5L* had a demotivated effect on GMECs. The effect of si-*EVI5L* was similar to miR-574-5p, and it also played a negative role in regulating the viability of GMECs. In brief, *EVI5L* promoted the growth of GMECs and downregulated their apoptosis.

Herein, WB was performed to investigate the effect of *EVI5L* on Bcl2 and Bax in GMECs. The results showed that the overexpression of *EVI5L* (pc3.1-*EVI5L*) increased the expression of Bcl2 but decreased Bax expression, while si-*EVI5L* had an antipodal impact (Figure 3G,H, Figure S1I,J).

### 3.4. EVI5L Increased the Milk Synthesis via PI3K/AKT–mTOR Signaling Pathway 

**Figure 4:**
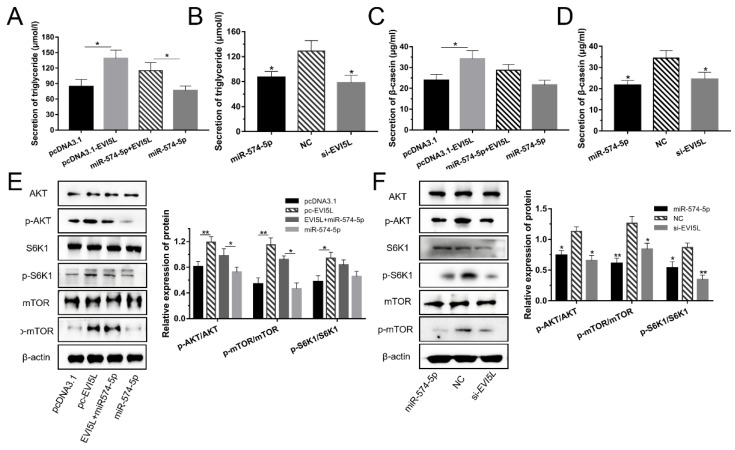
*EVI5L* increased the milk synthesis in GMECs via the PI3K/AKT–mTOR pathway. (**A**,**B**) The secretion of triglycerides in GMECs was measured with a detection kit. (**C**,**D**) The expression of β-casein in the cell-free supernatants by enzyme-linked immunosorbent assay kit. (**E**,**F**) WB assay was performed to detect the protein expression of the PI3K/AKT–mTOR pathway in GMECs. Pc-*EVI5L*: overexpression *EVI5L* plasmid; pcDNA3.1: control group; NC: negative control; si-*EVI5L*: small interfering RNA; miR-574-5p*+EVI5L*: miR-574-5p+pcDNA3.1-*EVI5L*. ** means significant difference (*p* < 0.01), * means significant difference (*p* < 0.05).

After the 24 h transfection of the plasmids in GMECs, *EVI5L* up-regulated the TAG expression and inhibited the function of miR-574-5p (Figure 4A,B). Figure 4C,D showed that the *EVI5L* overexpression plasmids (pcDNA3.1-*EVI5L*) augmented the expression of β-casein in contrast with pcDNA3.1. Si-*EVI5L* and miR-574-5p. which decreased the expression of β-casein.

The results of the WB assay are shown in Figure 4E,F; *EVI5L* activated AKT, S6K1, and mTOR. Compared with the pcDNA3.1 group, the AKT, mTOR and S6K1 phosphorylation ratios increased significantly in the *EVI5L* overexpression vector (pc-*EVI5L*)-treated group. For miR-574-5p +*EVI5L*, the AKT and S6K1 phosphorylation ratios were significantly higher than that of miR-574-5p. On the contrary, the si-*EVI5L* groups decreased AKT, S6K1, and mTOR phosphorylation. MiR-574-5p exerted the same effect as si-*EVI5L*.

### 3.5. CircRNA-006258 Promoted EVI5L Expression in GMECs

**Figure 5:**
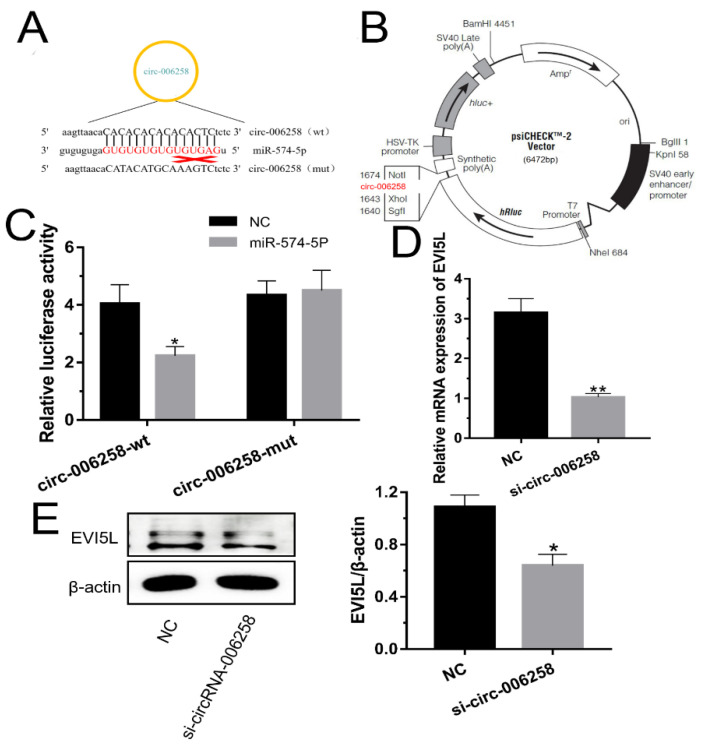
CircRNA-006258 promoted the EVI5L expression in GMECs. (**A**) circRNA-006258 existed complementary sequence (GCS) binding the seed sequence of miR-574-5p. (**B**,**C**) Contribution vector used to test the luciferase expression and the result of luciferase assay. (**D**) The analysis result concerning the mRNA expression of *EVI5L* by RT-qPCR. (**E**) Western blot analysis of *EVI5L* expression after 48 h transfection with circRNA-006258 and NC in GMECs. Dual-luciferase reporter vector of circRNA-006258: wild type: WT-circRNA-006258-psiCHECK2; mutant type: MUT-circRNA-006258-psiCHECK2; NC: negative control; si-circRNA-006258: small interfering RNA. ** means significant difference (*p* < 0.01), * means significant difference (*p* < 0.05).

On the basis of prior work, the target circRNA of miR-574-5p was predicted based on the circRNA library constructed previously. Through referring to this circRNA library and predicting by biological software, we found that there might be a sponge relationship between circRNA-006258 and miR-574-5p (Figure 5A).

To verify this conjecture, we built two types of dual-luciferase reporter vector of circRNA-006258: wild type: WT-circRNA-006258-psiCHECK2 (Figure 5B); and mutant type: MUT-circRNA-006258-psiCHECK2. By measuring the luciferase activity after transfection, we found that the double luciferase activity of the WT-circRNA-006258-psiCHECK2 and miR-574-5p group was significantly lower than that of the WT-circRNA-006258-psiCHECK2 and the negative control (NC) (Figure 5C). There was no significant change for the MUT-circRNA-006258-psiCHECK2 group. It is speculated that circRNA-006258 is capable of adsorbing miR-574-5p as a "sponge". As shown in Figure 5D, the mRNA expression of *EVI5L* in the si-circRNA-006258 group is notably lower than that of the NC group. The WB assay showed the same results (Figure 5E). It turned out that circRNA-006258 adsorbed miR-574-5p and promoted the expression of *EVI5L* in GMECs.

### 3.6. CircRNA-006258 Promoted GMEC Proliferation in GMECs

**Figure 6:**
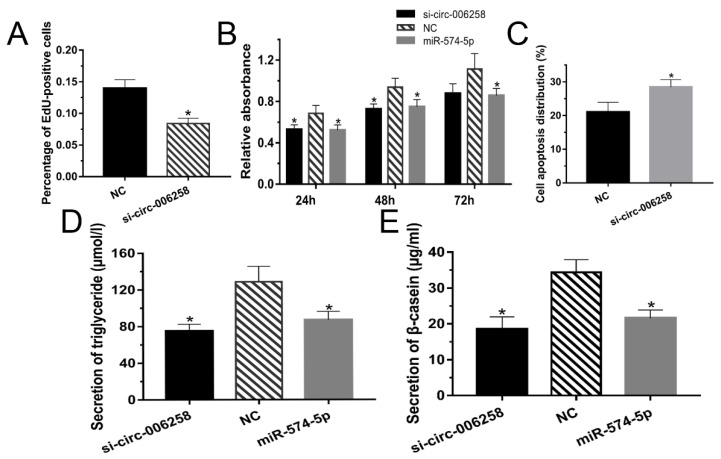
CircRNA-006258 promoted GMEC growth. (**A**) EdU was used to analyze the cell proliferation when the GMECs were cultured for about 24 h after transfection. (**B**) Cell viability of the GMECs was determined by CCK-8 when the cells were cultured for 24, 48, and 72 h after transfection. (**C**) Flow cytometry was performed to analyze cell apoptosis after transfecting for about 24 h. (**D**) The secretion of triglycerides in the GMECs was measured with a detection kit. (**E**) The expression of β-casein in the cell-free supernatants by enzyme-linked immunosorbent assay kit. NC: negative control; si-circRNA-006258: small interfering RNA. ** means significant difference (*p* < 0.01), * means significant difference (*p* < 0.05).

The cell proliferation was detected by the EdU kit. Figure 6A and Figure S1G indicate that the cell number of the si-circRNA-006258 group was less than that of the NC group. Cells were collected at specific times (24, 48, and 72 h) and tested by CCK-8 to examine the viability of the GMECs. Cell viability decreased significantly for all the time periods (Figure 6B). The cells apoptosis percentage of si-circRNA-006258 increased significantly compared with the NC (Figure 6C and Figure S1H). These results demonstrated that the circRNA-006258 promoted the proliferation of GMECs, enhanced cell viability, and inhibited their apoptosis.

### 3.7. CircRNA-006258 Promoted Milk Synthesis of GMECs 

Figure 6D showed that the triglyceride secretion of GMECs in the si-circRNA-006258 and miR-574-5p group was significantly blocked in contrast with the NC group. Moreover, the β-casein in GMECs after si-circRNA-006258 transfection was significantly reduced compared with the NC group (Figure 6E). Data indicate that circRNA-006258 promoted the synthesis of triglycerides and β-casein in GMECs.

### 3.8. CircRNA-006258 Regulated Cell Growth and Milk Synthesis 

**Figure 7:**
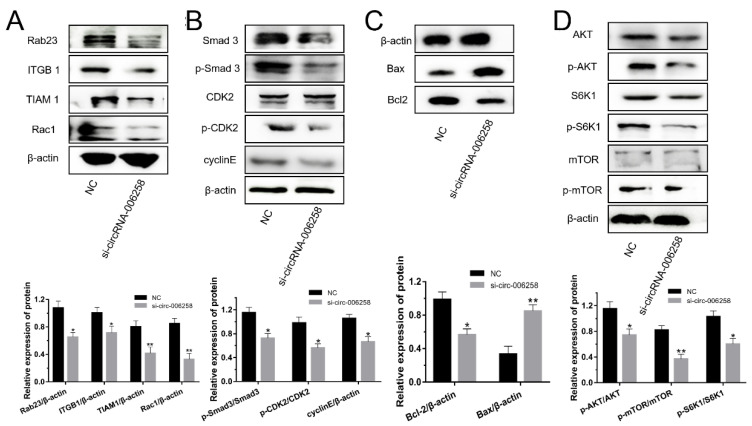
CircRNA-006258 promoted the milk synthesis of GMECs. (**A**) CircRNA-006258 influenced the protein expression of the Rab23/ITGB1/TIAM1/Rac1 pathway by WB. (**B**) Western blot analysis assessed the activation of circRNA-006258 on the TGF-β/Smad pathway in GMECs. (**C**) WB assay was conducted to investigate the protein expression of Bcl2 and Bax in the GMECs. (**D**) Investigating the effect of circRNA-006258 on the protein expression of the PI3K/AKT–mTOR pathway by WB assay in the GMECs. NC: negative control; si-circRNA-006258: small interfering RNA. ** means significant difference (*p* < 0.01), * means significant difference (*p* < 0.05).

From Figure 7A,B, for the cell cycle of GMECs, we found that si-circRNA-006258 inhibited Rab23/ITGB1/TIAM1/Rac1 protein expression, restrained the percentage of total protein and the phosphorylation of Smad3 and CDK2 in the TGF-β/Smad pathway and depressed the expression of cyclinE. As for regulating the cell growth of GMECs, si-circRNA-006258 inhibited the protein expression of Bcl2 and promoted the expression of the Bax protein (Figure 7C, Figure S1K). Thereby, for milk synthesis in GMECs, si-circRNA-006258 barred the phosphorylation of AKT, mTOR, and S6K1, and inhibited the PI3K/AKT–mTOR pathway (Figure 7D).

## 4. Discussion

Researchers found that the TGF-β/SMAD pathway could influence cell growth and regulate the cell cycle to transform G1 into S phase [30]. Complexes of cyclin-dependent kinases (CDKs) and cyclins the regulated cell cycle. CDK4/CDK6-cyclin D and CDK2-cyclin E separately altered the G1 and S phase of cells [31]. In our experiment, flow cytometry confirmed that *EVI5L* promoted S phase in GMECs, and the results of the WB assay for the protein of the cell cycle showed that *EVI5L* promoted the expression of CDK4, CDK2, cyclinD1 but inhibited cyclinE protein in GMECs. This is consistent with the report of Clurman B.E. [32]. Cells stopped at the G1 phase will go into G0 phase, which is known as the static condition: no growth, no fission and no migration. The cells in G0 phase will exaggerate the limit point and quickly return to the cell cycle and proliferation when they are stimulated by external signals such as serum and growth factors [33]. The hypophosphorylated and dephosphorylated of pRb appear in the G0 phase, while the Phosphorylation prevents cells from re-entering the cell cycle and phosphorylated pRb mediates G0/G1 transformation [34]. Beyond that, the Myc pathway takes the main role for cell cycle. Therefore, the effect of *EVI5L* on the Myc pathway in GEMCs is supposed to be explored and illuminated in the next stage.

Rab23 promotes hepatocellular carcinoma cell migration via the Rac1/TGF-β signaling pathway and promotes squamous cell carcinoma cell migration and invasion by regulating the integrin β1/Tiam1/Rac1 pathway [35]. Curcumin could inhibit SDF-1α-induced invasion through Rac1/PI3K/Akt signaling complexes in human esophageal carcinoma cells [35,36,37,38]. Hence, we speculated that these pathways might regulate the biological processes in GMECs. Therefore, we measured the related protein expression by WB. The function of *EVI5L* was brought into effect by Rab23/ITGB1/TIAM1/Rac1 in GMECs. Additionally, *EVI5L* affected the growth of the GMECs via Rac1-TGF-β/Smad and Bax, Bcl2. The activation of the PI3K/AKT–mTOR pathway by *EVI5L* accelerated the milk synthesis of the GMECs.

More and more studies have proved that non-coding RNA (ncRNAs) molecules are widely involved in udder development and the physiological activities of lactation. The competitive endogenous RNA (ceRNA) hypothesis is a new model for post transcriptional gene regulation. According to the hypothesis, the expressions of designated miRNAs are reduced by ceRNA [39]. Nowadays, a lot of evidence has attested that the form circRNA–miRNA–mRNA is important to explore the pathogenesis of tumors and other diseases. On the strength of this research, their targeted drugs are expected to be found. Building the circRNA–miRNA–mRNA template, various biological processes were better elucidated. Xiaotong Su et al. reported that circ-0070269/miR-182/NPTX1 played a significance role in hepatocellular carcinoma (HCC), and that this axis could serve as a potential therapeutic target [40]. Another research revealed that circ-016910 bound miR-574-5p to regulate cell physiology and milk synthesis via MAPK and PI3K/AKT–mTOR pathways in GMECs [11]. The reporters of the circRNA–miRNA–mRNA net are fewer in goat lactation performance. We found that circRNA-006258 directly bound miR-574-5p by the dual-luciferase reporter system. CircRNA-006258 relieved the inhibitory effect of miR-574-5p on *EVI5L* through sponging miR-574-5p. We established the network of circRNA-006258-miR-574-5p-*EVI5L* to fill the gap.

In conclusion, based on the result of preliminary laboratory research: miR-574-5p is differentially expressed in the colostrum and the peak lactation periods the target gene of miR-574-5p and ncRNA database were established by the laboratory, and miR-574-5p was selected as the research object. The targeting relationship between miR-574-5p and EVI5L was studied as well as circRNA-006258, to elucidate the molecular regulation mechanism of mammary epithelial cells in lactating goats. This study may contribute to determine the better regulation molecular mechanism of lactation performance in dairy goats.

## 5. Conclusions

**Figure 8:**
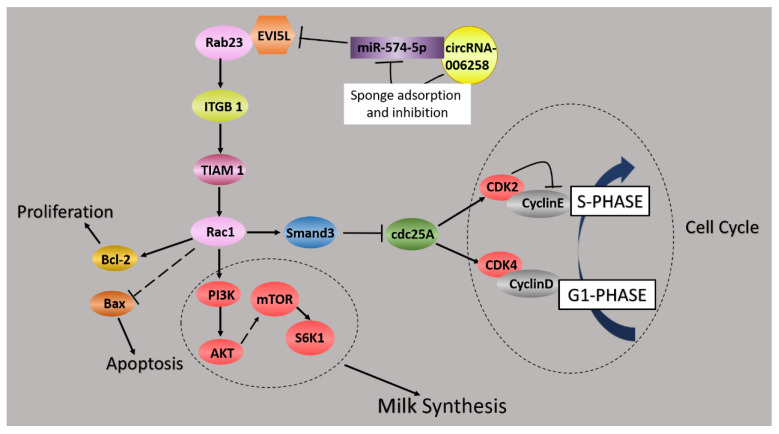
circ-006258/miR-574-5p/*EVI5L* regulates cell growth and the milk synthesis in GMECs.

In brief, our data indicated that circ-006258 was a sponge for miR-574-5p to regulate the growth and milk synthesis through *EVI5L* in the lactation period of GMECs. We founded the circRNA-006258/miR-574-5p/*EVI5L* axis and elucidated the function and the regulatory mechanism of this axis in the lactation period of GMECs (Figure 8).

## Figures and Tables

**Table 1:** Primer information for RT-qPCR.

Gene	GenBank Accession No.	Primer Sequences (5’→3’)
miR-574-5p		FORWARD: CGGTATGAGTGTGTGTGTGTGAG
		REVERSE: ATCCAGTGCAGGGTCCGAGG
		RT PRIMER: GTCGTATCCAGTGCAGGGTCCGAGGTATTCGCACTGGATACGACCACACA
U6		FORWARD: CTCGCTTCGGCAGCACA
		REVERSE: AACGCTTCACGAATTTGCGT
EVI5L	XM_018050954.1	FORWARD:TGTGTTCGTGCGGCTGATGC
		REVERSE:AGGTGGTGAGGAAGAGTGTGAGG
circRNA-006258	/	FORWARD:AGCGGCATCTCCACCATCTG
		REVERSE:GAAACACTTGGCTGGGCTGG
β-actin	XM_018039831.1	FORWARD: GATCTGGCACCACACCTTCT
		REVERSE: GGGTCATCTTCTCACGGTTG

**Table 2:** All of the main antibodies of Western blot (WB).

Name	Manufacturer	Product Number
β-actin	Beyotime, Shanghai, China	AA128
EVI5L	abcam, Cambridge, U.K.	ab243722
Rab 23	ABclonal, Wuhan, China	A7979
ITGB 1	Sangon Biotech, Shanghai, China	D120869
TIAM 1	ABclonal, Wuhan, China	A10252
Rac 1	ABclonal, Wuhan, China	A7720
Smad 3	ABclonal, Wuhan, China	A11388
p-Smad 3	ABclonal, Wuhan, China	Ao0554
CDK 4	Sangon Biotech, Shanghai, China	D120396
p-CDK 4	ABclonal, Wuhan, China	Ap0593
Cyclin D1	ABclonal, Wuhan, China	A11310
CDK 2	Sangon Biotech, Shanghai, China	D120395
p-CDK 2	Sangon Biotech, Shanghai, China	D155352
Cyclin E1	ABclonal, Wuhan, China	A14225
AKT	Cell Signaling, America	#9272
p-AKT	Cell Signaling, America	#9271
mTOR	Abway, Beijing, China	CY5306
p-mTOR	Boster, Wnhan, China	BM4840
Bcl 2	Abway, Beijing, China	CY6717
BAX	Abway, Beijing, China	CY5059
S6K1	BBI, Shanghai, China	D199437
p-S6K1	BBI, Shanghai, China	D151520

## Supplementary Materials

The following are available online at https://www.mdpi.com/2073-4425/11/7/718/s1, Figure S1: *EVI5L* and circ-006258 promoted GMEC proliferation in GMECs, Table S1: The validated primers used for psiCHECK-2 vectors, Table S2: EVI5L, circRNA-006258 and miR-574-5p sequences.
