# Supplementary material for: CircRNA-006258 Sponge-Adsorbs miR-574-5p to Regulate Cell Growth and Milk Synthesis via EVI5L in Goat Mammary Epithelial Cells
Source: Genes (Basel). 2020 Jun 28;11(7):718. doi: 10.3390/genes11070718 (PMC7397305; doi:10.3390/genes11070718)
Supplement: Supplementary file 1 [file genes-11-00718-s001.pdf]

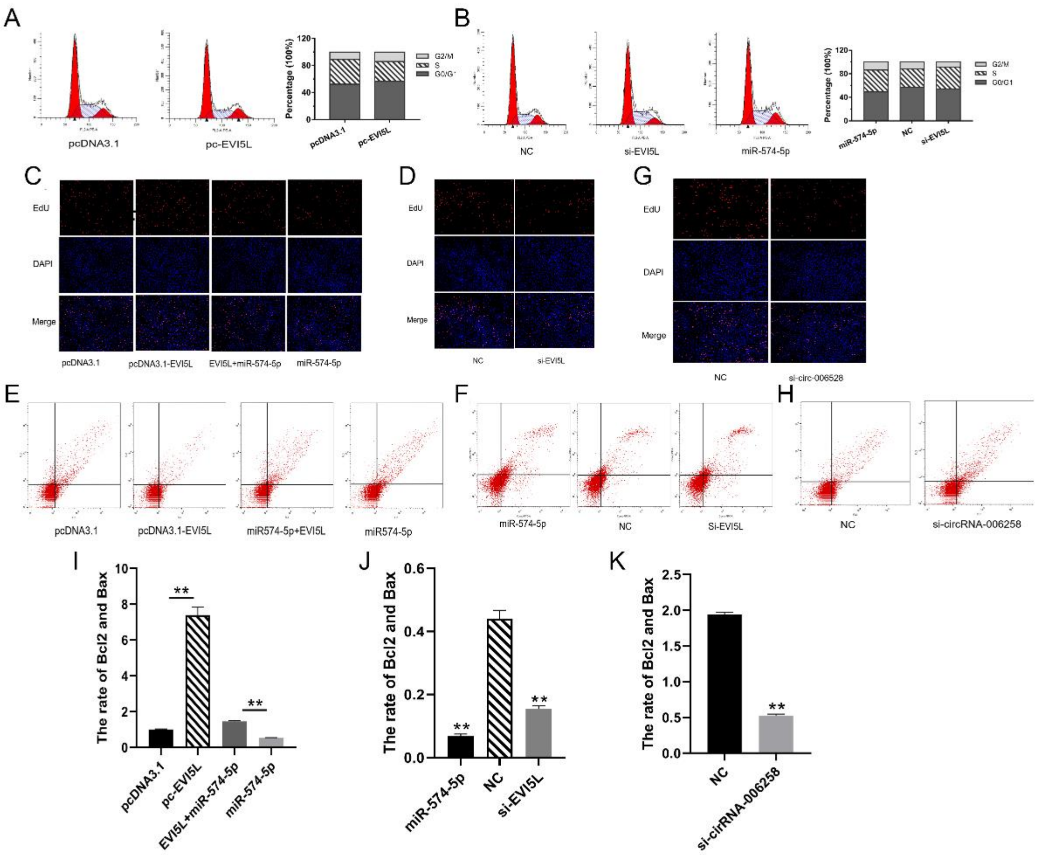

Figure S1: *EVI5L* and circ-006258 promoted GMEC proliferation in GMECs

Table S1 The validated primers used for psiCHECK-2 vectors

Name			Sequence (5'→3')
EVI5L	wild type	FORWARD	CACTCGAGCCTTCTCGGTGCTCCTGGAG
		REVERSE	ATGCGGCCGCATCTTTCCTGCCTGGGCGAC
	mutant type	FORWARD	CCCTCGAGGGACCTCATGACCCCCGG
		REVERSE	TAAAGCGGCCGCTGCTTTATTCCATCTTTCCTG
circ-006258	wild type	FORWARD	CGCTCGAGTGACCACATCACTCTGGGGC
		REVERSE	GCGGCCGCCAGTCTTGTGGGGCTGGGAA
	mutant type	FORWARD	CCCTCGAGGGATTTGCTTCAAGATAACCTGATGGG
		REVERSE	TAAAGCGGCCGCGGAAGCTGTTGCATGGAGAGC

Table S2 EVI5L, circRNA-006258 and miR-574-5p sequences

miR-574-5p	TGAGTGTGTGTGTGTGAGTGTGTG
EVI5L	TTCACAGACAGTGCTGTGACCTTACCCTGCTCACAGCTGAGGAGCCT<br>GACCACCATGGCGAGCCCCACTCTGAGCCCCGACTCCTCATCCCAGG<br>AGGCCCTGTCAGCCCCTACCTGCTCCCCCACCTCTGACTCCGAGAAC<br>CTCAGCCCCGATGAGTTGGAGCTGCTGGCCAAGCTTGAAGAGCAGA<br>ACCGGCTACTGGAGGCCGACTCCAAGTCCATGCGCTCCATGAATGGC<br>TCCCGGCGGAACAGCGGCTCCTCGCTGGTGTCCAGCTCCTCAGCCTC<br>CTCCAACCTGAGCCACCTGGAGGAGGACACGTGGATTCTGTGGGGCC<br>GGATTGCCAATGAGTGGGAGGAGTGGCGACGCCGGAAGGAGAAGCT<br>GCTGAAGGAGCTGATCCGCAAGGGCATCCCACACCACTTCCGGGCC<br>ATCGTCTGGCAGCTCCTGTGCAGTGCCACAGACATGCCGGTTAAGAA<br>CCAGTACTCAGAGCTGCTCAAGATGTCGTCCCCCTGCGAGAAGCTCA<br>TCCGCAGGGACATCGCCCGCACCTACCCAGAGCACGAGTTCTTCAAG<br>GGCCAGGACAGCCTAGGCCAGGAGGTCCTCTTCAATGTCATGAAGG<br>CCTACTCGCTGGTGGACCGGGAGGTGGGCTACTGCCAGGGCAGCGC<br>CTTCATCGTGGGCCTGCTGCTCATGCAGATGCCTGAGGAGGAGGCCT<br>TCTGTGTGTTCGTGCGGCTGATGCAGGAGTACCGCCTACGGGAGCTC<br>TTCAAGCCCAGCATGGCCGAGCTGGGGCTCTGCATCTACCAGTTTGA<br>ATACATGTTACAGGAGCAGCTCCCGGACCTGAACACCCACTTCCGCT<br>CCCAGAGCTTCCACACGTCCATGTATGCCTCGTCCTGGTTCCTCACAC<br>TCTTCCTCACCACCTTCCCACTGCCCGTCGCCACCCGTGTGTTTGACA<br>TCTTCATGTACGAGGGCCTGGAGATCGTCTTCCGGGTGGGCCTCGCC<br>CTGCTGCAGGTGAACCAGACGGAGCTGATGCAGCTGGACATGGAGG<br>GGATGTCCCAGTACTTCCAGAGGGTGATCCCCCATCAGTTCGACAGC<br>TGTCCAGATAAGCTGATCCTCAAGGCTTACCAGGTCAAGTACAACCC<br>CAAGAAGATGAAGAGGCTGGAGAAGGAGTATGCGGCCATGAAGAG<br>C AAGGAAATGG AGGAGCAGAT TGAGATCAAGAGGCTTCGGA<br>CGGAGAACCGGCTCCTGAAGCAGCGGATCGAGACCCTGGAGAAGGA

	GAGCGCTGCTCTGGCTGATAGGTTAATACAGGGGCAGGTGACGCGC<br>GCACAGGAGGCAGAGGAGAACTACGTCATCAAGCGGGAACTGGCTG<br>TGGTGCGGCAGCAGTGCAGCTCGGCGGCCGAAGACCTGCAGAAGGC<br>GCAGAGCACAATCCGGCAGCTGCAGGAGCAGCAGGATAACCCGCGC<br>CTCACCGAAGACTTTGTGGCCCACCTGGAGACGGAGCTGGAGCAGTC<br>ACGGCTTCGGGAGACCGAGACCCTGGGGGCACTTCGGGAGATGCAG<br>GACAAGGTTCTGGACATGGAAAAGAGGAACAGCTCGCTGCCCGACG<br>AGAACAACGTGGCTCGGCTTCAGGAGGAGCTGAAGGCGCTCAAGGT<br>TCGGGAGGGTGAGGCGGTGGCCTCGGCGCGGGAGCTGAAGCTGCAG<br>CTGCAGGAGCTCTCGGACACCTGGCAGGCCCACCTGTCCCGCGGTGG<br>CCGCTGGAAGGAGTCCCCGCGGAAGTTGGTCCTGGGCGAGCTGCAG<br>GACGAGCTGATGAGCGTGCGGCTGCGCGAGGCCCAGGCGCTGGCCG<br>AGGGTCGAGAGCTGCGGCAGCGCGTGGTGGAGCTCGAGACGCAGGA<br>CCATATCCACCGCAACCTGCTGAACCGTGTGGAGGCTGAGCGCGCGG<br>CGCTGCAAGAGAAGCTGCAGTACCTGGCGGCGCAGAACAAGGGGCT<br>GCAGACGCAGCTCAGCGAGAGCCGCCGCAAGCAGGCGGAGGCCGAG<br>TGCAAGAGCAAGGAGGAGGTGATGGCCGTGCGCCTGCGGGAGGCGG<br>ACAGCATGGCGGCGGTGGCCGAGATGCGGCAGCGCATCGCCGAGCT<br>GGAGATCCAGAGAGAGGAGGGCCGCATCCAGGGCCAGCTGAACCAC<br>TCGGACTCGTCGCAGTACATCCGCGAGCTCAAGGATCAGATCGAGG<br>AGCTGAAGGCCGAGGTGCGGCTGCTGAAGGGTCCGCCGCCCTTTGA<br>GGACCCGCTGGCCTTCGACGGGCTGGGCCTGGCGCGGCACCTGGAC<br>GAGGACTCACTGCCGTCGTCCGACGAGGAGCTGCTGGGCGTGGGCG<br>TGGGCGTGGGCGCCGCGCTGCAGGATGCGCTCTACCCGCTGTCCCCG<br>CGCGACGCGCGCTTCTTCCGCCGGCTGGAGAGGCCGGCCAAGGACA<br>GTGAGGGCAGCTCCGACAGCGACGCCGACGAGCTGGCTGCGCCCTA<br>CAGCCAGGGCCTGGACAACTGAGGCCGTGCCCCGCGTGCTCGGAGC<br>CAGGGCGCGGGGGTAGGCGGGTGGGCCGCCGCCAGCCCGCTCCTGC<br>GTGGGCGCCAGCTCTTCACTGACTCCACGCCCTCGGCGGTTTCACCC

	ACCCAGAGGGCCAGGCCCAGCACACAGCTGGGCACAGAGGGCAGGG<br>CCGGAGGCTGGGGATCCTCACAGTGCCCTGGCAGGCCCACCCTCCCC<br>AGCAGTGTTTACCCATCTTGGTCCGTTCCCTTCTCGGCCCGTTTACCT<br>GGAAGGTCTGCCTCTCTTAACAGCCAGGGCCAGGAGGACCCAAGGG<br>TCCCAGGACTGCACCAACGGACGCTCCCTCTCCTAGCTGGCCTCCTC<br>CTGGGACAACAGCGGCCCTGCCGGGAAGGGCCCAGGCTCTGGGGCA<br>GGCCTGAGGAAGAAGGTGCCCCTGTAGGCCAGACTTTACCACCCATG<br>AAGGCCCTGGGGACCCTTGGCCTCTGGAGATAGATGGCCAGGCACT<br>GCCTGGTGCAGGCATTGTGGCCCCTGCAACAGAAACCAAAGTCCCCC<br>CTACTGAGTGCAGGGCTGCCAGGGTGTGAGCGGCTAGCTGGAACCA<br>GCCAGGGCCTGGGCAGTACCTGTCCCTATTTCCTAGAGGCCAGAGCA<br>GCTGTAAGGAAGTGGAGAGACCTCCCCCTCCCCAGCACCCCACCTCT<br>TCCTCTGTGGCAGCCCCATCACCCCAGCTTGGCCAGTGACGGGGGGA<br>GAGGAGCTCCCCATGCAGGCTAGCCTCCTCTCCAGGGGATCCCCAGC<br>CTCCAAGTCCCTTCCTCCTCTGTATCCCTAGCCCCACCCTCCAGCCCC<br>AGGGGAGGAGGCCTCCTGGCTGAGTTCCCCCCACAACCTGCTCACAG<br>TGGTCCCGTGGTCTCTGCTGAGTCAGAGCCAAGAACGGTGCCAAAGT<br>CCAACGATGCCCCCAGATCCAACCCCCCGGGACCTCATGACCCCCGG<br>GCTGACCTTCTCGGTGCTCCTGGAGCCTAGAACAGCATCACAAACAG<br>TCCCCCTGGCGCTTCCTGGGGTGGAGATCCATGCTCAGTCCCCCCTTC<br>CCGGTCGCCCACTCAGTGTTACAAAGCCTGGGGACCAGGGTGGGCAT<br>CCCAGGGGCTCACCCACATGCCCCCCGCACTGCCCACCACCATTTTG<br>CACACTGCCTGTTCACACTCCACATGTCGCCCAGGCAGGAAAGATGG<br>AAAATAAAGTGTATTTACACAGTCA

circ-006258	GCAGTGGCGTGGAACACGCTGGCCAGGTACAGGTCCTTCTTGCCCGA<br>CATGCGAGACAGGTCTGCCTTGTTCTTGTCGATGGCCTCGGTCAGAC<br>CCAGCCCAGCCAAGTGTTTCTGTAGAACCAGGTGGATGATGGTGAAG<br>CCATGGTAGGCAAAGTTCCTAGCCCCTTACCCTCCACCCACACCACC<br>CACTGTCTCTCTTCTTCTCTGAGCAGAAGTCACCATTCCCTGGCCACC<br>TTCTGTGTGCCAGACATGGTGTGGAAACTGCAAACCACTTTCCTAAT<br>TCTTGACAGCCCTGCAAGAGCTTCCAATCACCACTTCACAGATGAGG<br>AAACCAAGACTCAGGGGGAAAAGGCAGAATATATGCCTGGACCCAC<br>TGAGCAGTCTTGACCTGTGGTATGAGTAGTAGACAGCCAGCATCGGC<br>CTCCCCTGGGGGCTTATATGAAATGCACGTTTTCTGCTCCAGCCCAG<br>AGCCTCTGAATCACAGTGTCTGTAGGTAGACCAGGAACCCTTCTTAA<br>CCAACTCTCCAGGTAATTCTTGACACACTCATGTTTGAGAAAGACTG<br>ACCTAAGGACAGCTCTGACCACATCACTCTGGGGCTTAAAATCTTTC<br>CAGAGTGATATGAAGGCTGGGATTTGCTTCAAGATAACCTGATGGGT<br>GTGATAGTTGATGTTGAGTGATGGATACATGAGGGTTCCTTACATTA<br>ATCTATTTTTAATATATGTTAATGTATTTCCATAGTATCTCAAAAGTT<br>AACACACACACACACACTCTCTCTCTCTTCTCTCTCTCTTTCCAAG<br>GCTCTGCACAGCCCCTGGAAGCCAAGGACAAGCTCTTTCCTAGGAAG<br>TGTGCTTGCATCACAGTTTCACCCCTAGCATGTACCACTCTGCTCTCC<br>ATGCAACAGCTTCCCTGAAACTGCCTCCTCAATAGCATCTTTGTGTCT<br>ACCCTGTCTCTGTAAGGCCCTTTAGACGCCACGTCTTCTGGGGAGCC<br>TTCCCAGCCCCACAAGACTGGAGCCACGCAGCCCTGGCCTCGCTCTA<br>TCAGTATATGGTCCTGTTGTCTCTCCCACCTGGCTCGGTGCTCCCTCA<br>AGGCAGGGCCCATGTTTGGGGCTCACCCTGCTCTCAGTGCAGAAGAG<br>GCGGTGGTAAAGAACTGTCGGCTGAGTGAACAGCTGCCCTCAGGGC<br>CCAGCTTCTGGAGGACAGTGCACCTCCCCAGCATGGCCAAACCGGCC<br>TGATACCTAGGGGCCTGAGAGTCCAGGTGTCCCGAGAGAGGCATTC<br>GAGGCCCAGCTATTCCAGTCCCCATGACTTTGCACAAATTACCACTT<br>GGAATTTTCCACCTCCATCTACTGTAGGGTGGTAGACAGTGTGTCCA<br>CCCAGCGTGCCAGGAAAACACGGGAGAAAGAAGACGAGTCAGGTG<br>ATGGCAACTGCAGAACGGGCTGGGGGAGTGTCAGAACAAGCCCAGG<br>AGGGAGACGGGTGTGGCTTCTCTCTGGAAACCTCATGCTGTCTACCG<br>CACAGACCACACAAGTTCTACCAGCCTCCAGCTTGG<br>AGTCTAAGCCCCATCTCATCTCAAGCTTTCTCCTCCACACTCCCTCAG<br>CAGCAAGTCTGAATCAATTCTGCAGGCAAGCGATCACCTACAGAAA<br>CGTTTCTGGAACCTAAGGGAGGAATGAGATGAAAGTTTTCCTTTTTG<br>AACAGAGAAAAAAAAAAAAATCTCTCCTGATTGACAGAAAGGCCAC<br>CCTGAGTCTCCACCTGAGAACTGCAGCCCCAGCACTTACTATCCAGG<br>GTAACCTGAGCAAGTCACTGCTTCTCTCTGAGCTGCTGCCCACCACC<br>CAGGGTGGTCATGAGGGGGAAACGATGCTTAGCAGGTTGGTTAAGT<br>TTCCAACCAAGTGACTCAGAGCTGAAAGCTATTATTAGTCTTACTAG<br>TGCATGGGGAACAATCCTCCAGCTTGAATAGTGGAGGCATACCCTAG<br>AGACATTGCGGGTTTGGTTCCAGACCACCACAATGAAGTGAATATTG<br>CAATAAGTGAGTCACACAAATTTTTTGCTTTCCCGATGCATATAAAC

	GTTATGTTTGCACTATAGTCTATCAAGTCTGCAACAGCATTATCCCTT<br>AAAACAATGTATGTACCTTAATTAAAAATGTTTCATTGCTAAAAATG<br>CTAACCATCATCTGAGTCTTTAGGGAGCTGTAATCTTTTGCTGGTGGA<br>GGGTTTGAAATATTGAGAGAATTACCAAAATGGGACCCAGAGACAT<br>GAAATGAGCAAACGCTGTTGGAAAAGTGGAGCCAGACTTGCTTGAT<br>GTAGAGTTGCCATAAATCTTCAAAAATTTGTAAAAAAAAAAAAAATTG<br>CAATAAAGAAAAGCACGATAAAATGAGGTATGCCTACACCTTGTGC<br>TAAGAGGCAGCACAATGTAAGATTAAGGACATGGCTTGATGCTAAA<br>TGTCTGAGTTAGTGACTGTATGACTCTGGATAAGTTATTCAACTTTTC<br>TGAGCCTCAGTTTCCTCATCTGTAAGATAAGGCTGATAGTCCTGCCT<br>AATATGGTGTTGAGGATTAAGAATTTAACACATGCAGAGTGCTTAGG<br>AAGTGTGCCTGGCACACAGCAGGTGCTCAATTATACTTAAGTATTGA<br>TGTTGTCACCATCTTCCCAAAGGCAGGGACCCATGCCCCCCTCCCTT<br>GGCACAGCCCAGTACTGCCTGGACACCCAGAGGGGCTCAGGACAGA<br>CCCGCTGAGCTGGTGACAGACCACCCCCCTGCCCAACTCTAAGCCCT<br>GCAGGCCCCTGAGTTGGGCCTCCCCTCACCTGCAGGTCGTGGGTCAC<br>CTCCACGACTCCCTTGGGCAGGGAGATGGCTACAGCCTTCTTCTGCA<br>TCTTGCCCATCCAGACCTTCAGCTGCTCTTTGGTCAGCAGCTTTTCCA<br>GGCGCTCAAGGGGCTCCACGTGGTGGGGCATGATGATGATGAGGCT<br>GGACAGCTTGTGCGCCAGCGGCATCTCCACCATCTGCAGCTTCTCCT<br>TCTCGTCGTCATAGTA
